# MPTP-driven NLRP3 inflammasome activation in microglia plays a central role in dopaminergic neurodegeneration

**DOI:** 10.1038/s41418-018-0124-5

**Published:** 2018-05-21

**Authors:** Eunju Lee, Inhwa Hwang, Sangjun Park, Sujeong Hong, Boreum Hwang, Yoeseph Cho, Junghyun Son, Je-Wook Yu

**Affiliations:** 1 Department of Microbiology and Immunology, Institute for Immunology and Immunological Diseases, Brain Korea 21 PLUS Project for Medical Science, Yonsei University College of Medicine, Seoul, Republic of Korea; 2 Doping Control Center, Korea Institute of Science and Technology, Seoul, Republic of Korea

**Keywords:** Inflammasome, Inflammasome, Neurological disorders

## Abstract

Parkinson's disease (PD) is a progressive neurodegenerative disease characterized by the loss of dopaminergic neurons in the substantia nigra (SN) and the reduction of dopamine levels in the striatum. Although details of the molecular mechanisms underlying dopaminergic neuronal death in PD remain unclear, neuroinflammation is also considered a potent mediator in the pathogenesis and progression of PD. In the present study, we present evidences that microglial NLRP3 inflammasome activation is critical for dopaminergic neuronal loss and the subsequent motor deficits in the 1-methyl-4-phenyl-1,2,3,6-tetrahydropyridine (MPTP) mouse model of PD. Specifically, NLRP3 deficiency significantly reduces motor dysfunctions and dopaminergic neurodegeneration of MPTP-treated mice. Furthermore, NLRP3 deficiency abolishes MPTP-induced microglial recruitment, interleukin-1β production and caspase-1 activation in the SN of mouse brain. In primary microglia and mixed glial cell cultures, MPTP/ATP treatment promotes the robust assembly and activation of the NLRP3 inflammasome via producing mitochondrial reactive oxygen species. Consistently, 1-methyl-4-phenyl-pyridinium (MPP^+^) induces NLRP3 inflammasome activation in the presence of ATP or nigericin treatment in mouse bone-marrow-derived macrophages. These findings reveal a novel priming role of neurotoxin MPTP or MPP^+^ for NLRP3 activation. Subsequently, NLRP3 inflammasome-active microglia induces profound neuronal death in a microglia-neuron co-culture model. Furthermore, Cx3Cr1^CreER^-based microglia-specific expression of an active NLRP3 mutant greatly exacerbates motor deficits and dopaminergic neuronal loss of MPTP-treated mice. Taken together, our results indicate that microglial NLRP3 inflammasome activation plays a pivotal role in the MPTP-induced neurodegeneration in PD.

## Introduction

Parkinson's disease (PD), the second most common neurodegenerative disorder, is characterized by a progressive degeneration of dopaminergic neurons in the substantia nigra (SN) and a subsequent reduction in the striatal concentration of dopamine, which leads to motor impairment [1, 2]. Although the precise etiology of PD remains unclear, the aggregation of α-synuclein in dead or dying dopaminergic neurons has been reported [3, 4] and is considered a primary cause of dopaminergic neuron degeneration. However, the molecular mechanisms underlying the formation and neurotoxicity of α-synuclein aggregates have not yet been elucidated [5].

In addition to the potential role of α-synuclein in PD pathogenesis, neuroinflammation has also been implicated in the loss of dopaminergic neurons [6, 7]. To this end, increased pro-inflammatory cytokine levels have been found in striatal dopaminergic regions or cerebrospinal fluid of PD patients [8, 9], and in the SN in an animal model of PD [10]. Additionally, activated microglia might contribute to neuronal damage via the release of pro-inflammatory cytokines and neurotoxic products [11]. Further, histological data revealed an increased number of activated microglia in the SN of PD patients, concurrent with the degeneration of dopaminergic neurons [12, 13]. Hence, the regulation of neuroinflammation mediated by microglia might provide a potential therapeutic strategy to mitigate the progression of PD.

Inflammasome is a caspase-1-activating multi-protein complex comprised of sensor molecules such as NOD-like receptor family, pyrin domain-containing 3 (NLRP3), apoptosis-associated speck-like protein containing a caspase recruitment domain (ASC) and procaspase-1 [14]. In microglia, NLRP3 inflammasome can be assembled upon stimulation with accumulated endogenous metabolites such as fibrillar amyloid β and 25-hydroxycholesterol [15, 16]. The assembly of an inflammasome promptly results in the activation of caspase-1 and the subsequent secretion of interleukin (IL)-1β, which might play a major role in the initiation of pro-inflammatory responses around microglia. Recent studies demonstrated a significant contribution of NLRP3 inflammasome to the progression of Alzheimer's disease (AD), the most common neurodegenerative disease [15, 17]. Amyloid β, a risk factor for AD pathogenesis, could promote the activation of the NLRP3 inflammasome pathway in microglia [15], and NLRP3 deficiency clearly attenuates AD phenotypes, including the loss of spatial memory, in aged APP/PS1 mice [17].

Of particular interest, fibrillar α-synuclein was shown to induce the secretion of IL-1β from peripheral blood mononuclear cells and microglia [18, 19]. Furthermore, a recent study revealed that caspase-1 deficiency significantly reduces dopaminergic neuronal death of mice following intoxication with 1-methyl-4-phenyl-1,2,3,6-tetrahydropyridine (MPTP), a compound commonly used in animal models to induce PD [20]. Interestingly, Wang et al. showed that caspase-1 could process α-synuclein into a truncated, aggregation-prone form that facilitates its aggregation [21]. Furthermore, blocking the IL-1 receptor by IL-1Ra in an animal study significantly attenuated the degeneration of dopaminergic neurons [22, 23]. These recent studies suggest that caspase-1 and the inflammasome pathway are potentially associated with PD pathogenesis or progression. Nonetheless, the mechanisms underlying the contribution of inflammasome signaling to dopaminergic neuron death remain largely unknown. In the present study, we provide evidences that neurotoxin MPTP activates the NLRP3 inflammasome in microglia, and that the NLRP3 inflammasome-activated microglia plays a pivotal role in the neurodegeneration associated with PD.

## Results

### NLRP3 deficiency alleviates motor dysfunctions and dopaminergic neuronal loss in MPTP-treated mice

**Fig. 1:**
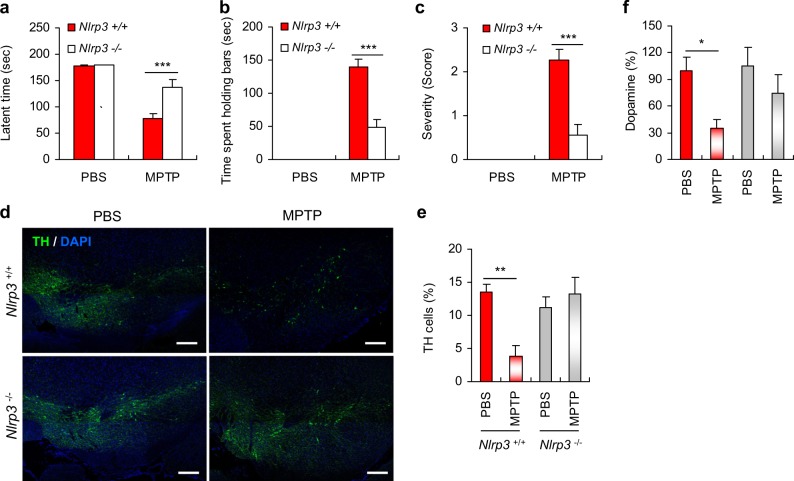
NLRP3 deficiency attenuates motor dysfunctions and dopaminergic neuronal loss in MPTP-treated mice. **a**–**c** *Nlrp3*^+/+^ or *Nlrp3*^−/−^ mice were administered with PBS or MPTP (40 mg/kg) for 5 days and then examined for motor activity impairment as described in the Methods. **a** The latent time to hold the bar in the string test 24 h post the final PBS or MPTP administration (*Nlrp3*^+/+^ mice, PBS (*n* = 6), MPTP (*n* = 10); *Nlrp3*^−/−^ mice, PBS (*n* = 6), MPTP (*n* = 11)). **b** The latent time to hold the bar in the catalepsy test 1 h post the final PBS or MPTP administration. (PBS (*n* = 6), MPTP (*n* = 11)). **c** The severity of hindlimb clasping in PBS- (*n* = 6) or MPTP-treated (*n* = 11) mice 1 h post the final PBS or MPTP administration. **d** Representative immunofluorescence image of the fixed brain sections containing SN of PBS- or MPTP-treated *Nlrp3*^+/+^ or *Nlrp3*^−/−^ mice after staining with anti-TH antibody (green). DAPI represents the nuclear signal (blue). Scale bars, 200 μm. **e** Quantification of relative TH-positive cells per DAPI in confocal images of PBS- or MPTP-treated *Nlrp3* ^+/+^ or *Nlrp3*^−/−^ mice (*n* = 4). **f** Quantification of striatal dopamine levels of PBS- or MPTP-treated *Nlrp3*^+/+^ or *Nlrp3*^−/−^ mice as determined by HPLC-MS/MS (*Nlrp3*^+/+^ mice, PBS (*n* = 11), MPTP (*n* = 11); *Nlrp3*^−/−^ mice, PBS (*n* = 11), MPTP (*n* = 10)). Data were expressed as the mean ± SEM. Asterisks indicate significant differences (*P < 0.05, **P < 0.01, ***P < 0.001)

To induce PD-like symptoms in mice, we administered intraperitoneal injections of MPTP to mice as previously described [24]. The administration of MPTP caused a significant impairment of motor activity as determined by a string test (Fig. 1a). Likewise, MPTP-treated mice exhibited a marked increase in the cataleptic response (Fig. 1b). The MPTP-induced motor dysfunctions were significantly attenuated in *Nlrp3*-deficient mice compared with *Nlrp3*-expressing wild type (WT) mice (Fig. 1a,b). Moreover, the MPTP-induced hindlimb clasping behavior in WT mice was markedly reduced in *Nlrp3*-knockout mice (Fig. 1c and Supplementary Fig. 1a). In accord with these observations, MPTP intoxication caused a severe truncal dystonia in WT mice, but the observed effects were reduced in *Nlrp3*-deficient mice (Supplementary Fig. 1b).

Next, we observed dopaminergic neurons stained with an anti-tyrosine hydroxylase (TH) antibody, in the SN of the mouse brain following MPTP administration. As expected, MPTP administration caused a significant reduction in the TH-positive dopaminergic neurons in the SN region of WT mice (Fig. 1d,e). Notably, the MPTP-induced loss of dopaminergic neurons was significantly protected by the NLRP3 deficiency (Fig. 1d,e). Consistent with the loss of TH-positive neurons, MPTP treatment resulted in a robust reduction of Nissl-positive neurons in the SN of WT mice, but not of *Nlrp3*-knockout mice (Supplementary Fig. 2). Furthermore, a significant reduction of striatal dopamine levels was observed in the MPTP-treated WT mice, but not in *Nlrp3*-deficient mice (Fig. 1f). These data indicate that NLRP3-mediated signaling is closely associated with dopaminergic neurodegeneration in the SN of MPTP-treated mice.

### NLRP3 deficiency abrogates MPTP-induced microglial recruitment and caspase-1 activation in the SN of mice

**Fig. 2:**
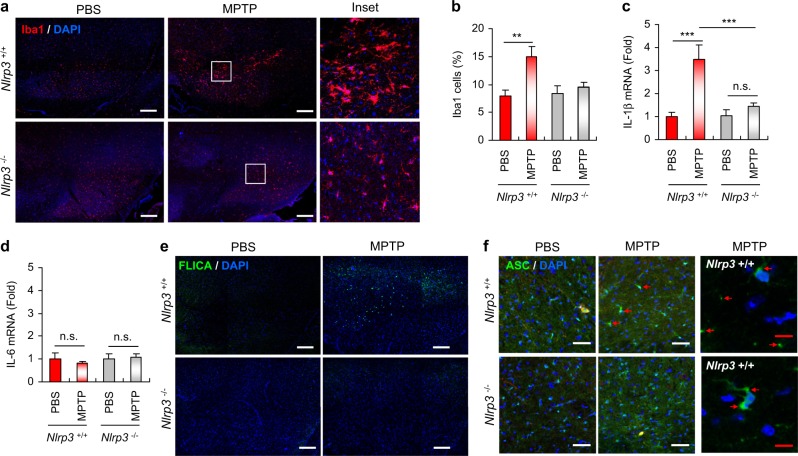
NLRP3 deficiency attenuates MPTP-induced microglial recruitment and caspase-1 activation in the SN of mouse brain. **a** Representative immunofluorescence image of the SN in mouse brain sections stained with anti-Iba1 antibody (red). DAPI represents the nuclear signal (blue). Scale bars, 200 μm. Magnified immunofluorescence images of the boxed areas in the middle panel are displayed in the right panel. **b** Quantification of relative Iba1-positive cells per DAPI in confocal images of PBS- or MPTP-treated *Nlrp3*^+/+^ or *Nlrp3*^−/−^ mice (*n* = 4). **c**, **d** Quantification of IL-1β (**c**) or IL-6 (**d**) mRNA levels in the SN of PBS- or MPTP-treated *Nlrp3*^+/+^ or *Nlrp3*^−/−^ mice. (*Nlrp3*^+/+^ mice, PBS (*n* = 9), MPTP (*n* = 9 (**c**) or 8 (**d**)); *Nlrp3*^−/−^ mice, PBS (*n* = 7), MPTP (*n* = 9)). **e** Representative immunofluorescence image of brain sections containing SN stained with FLICA. DAPI represents the nuclear signal. Scale bars, 200 μm. **f** Representative immunofluorescence image of substantia nigra *pars reticulata* (SNr) regions of PBS- or MPTP-treated *Nlrp3*^+/+^ or *Nlrp3*^−/−^ mice stained with anti-ASC antibody (green). DAPI represents the nuclear signal. Scale bars (white), 50 μm. Enlarged immunofluorescence images of SNr regions of MPTP-treated *Nlrp3*^+/+^ mice were displayed in the right panel. Scale bars (red), 10 μm. Red arrows indicate ASC specks. Data were expressed as the mean ± SEM. Asterisks indicate significant differences (**P < 0.01, ***P < 0.001, n.s. not significant)

Next, we examined whether MPTP intoxication could promote inflammation in the SN of mouse brains in our experiment. Interestingly, MPTP treatment resulted in a significant increase in the population of microglia, as determined by microglia-specific Iba1 staining, in the SN of WT mice, but not in the SN of *Nlrp3*-deficient mice (Fig. 2a,b). Furthermore, an activated microglia morphology was observed in WT mice following MPTP treatment, but not in *Nlrp3*-knockout mice (Fig. 2a, right panel).

We then checked IL-1β mRNA levels in the SN of MPTP-treated mice. In accord with the observed increase in microglial recruitment, MPTP administration caused a significant elevation in IL-1β mRNA expression in the SN of *Nlrp3*^+/+^ mice, but not in *Nlrp3*^−/−^ mice (Fig. 2c). Conversely, the administration of MPTP did not induce increases in IL-6 levels in the SN in either of the mouse strains (Fig. 2d). Then, we examined whether MPTP administration promoted the activation of inflammasome signaling in the SN by monitoring the presence of active caspase-1 using an active caspase-1-specific fluorochrome-labeled inhibitor of caspases (FLICA) probe. Notably, active caspase-1-containing cells were markedly elevated in the SN of WT mice, but were observed at a much lower frequency in *Nlrp3*-knockout mice following MPTP administration (Fig. 2e). The formation of inflammasome adapter ASC specks can be used as a unique hallmark of inflammasome activation in mouse model [25]. Intriguingly, MPTP injection caused a robust formation of ASC specks in the substantia nigra pars reticulata regions of WT mice, but much less of *Nlrp3*-deficient mice (Fig. 2f and Supplementary Fig. 3).

### MPTP mediates NLRP3-dependent caspase-1 activation and interleukin-1β production in microglia and mixed glial cells

**Fig. 3:**
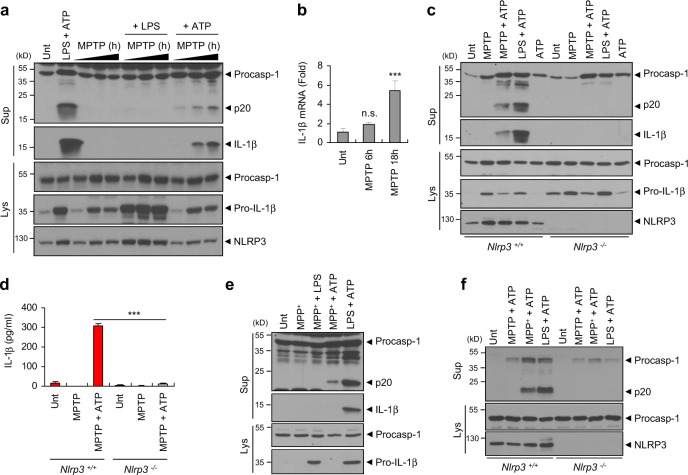
MPTP or MPP^+^ treatment promotes the activation of the NLRP3 inflammasome. **a** Immunoblots from mouse mixed glial cell cultures untreated (Unt) or primed with LPS (0.25 μg ml^−1^, 3 h), followed by ATP (2.5 mM, 40 min), or treated with MPTP (40 μM) for 6, 12 or 18 h in the presence of LPS (final 3 h) or ATP (final 40 min). **b** Cellular levels of IL-1β mRNA in mouse mixed glial cells untreated or treated with MPTP (40 μM) for 6 or 18 h (*n* = 4). **c** Immunoblots from *Nlrp3*^+/+^ or *Nlrp3*^−/−^ mouse microglia treated with MPTP (40 μM, 16 h), followed by ATP treatment (2.5 mM, 30 min), or primed with LPS (0.25 μg ml^−1^, 3 h), followed by ATP (2.5 mM, 30 min). **d** Quantification of IL-1β in the culture supernatants of *Nlrp3*^+/+^ or *Nlrp3*^−/−^ mice microglia treated with MPTP (40 μM, 16 h), followed by ATP treatment (2.5 mM, 30 min) (*n* = 3). **e** Immunoblots from mouse BMDMs treated with MPP^+^ (40 μM, 16 h), followed by ATP (2.5 mM, 30 min) or LPS treatment (0.25 μg ml^−1^, 3 h), or primed with LPS (0.25 μg ml^−1^, 3 h), followed by ATP (2.5 mM, 30 min). **f** Immunoblots from *Nlrp3*^+/+^ or *Nlrp3*^−/−^ mice BMDMs treated with MPTP or MPP^+^ (100 μM,6 h), followed by ATP (2 mM, 45 min), or primed with LPS (0.25 μg ml^−1^, 3 h), followed by ATP (2 mM, 45 min). **a**, **c**, **e**–**f** Culture supernatants (Sup) or cellular lysates (Lys) were immunoblotted with the indicated antibodies. Data were expressed as the mean ± SEM. Asterisks indicate significant differences (***P < 0.001, n.s. not significant)

To elucidate the molecular mechanisms underlying the contribution of NLRP3 to the MPTP-induced PD phenotypes, we first examined the potential of MPTP to directly activate caspase-1 in the brain glial cells. In mouse primary mixed glial cell cultures, MPTP treatment alone did not induce the activation of caspase-1 or the secretion of IL-1β as determined by the detection of active caspase-1 (p20) and mature IL-1β in the cell culture supernatants (Fig. 3a). Intriguingly, priming with MPTP followed by treatment with ATP, but not lipopolysaccharide (LPS), promoted a robust processing of caspase-1 and IL-1β in the immunoblots of the cell culture supernatants (Fig. 3a). Furthermore, prolonged MPTP treatment caused the marked elevation of pro-IL-1β mRNA expression (Fig. 3b), whereas the increase in IL-6 transcripts was less apparent than that of pro-IL-1β in mixed glial cells (Supplementary Fig. 4a).

We then verified this unexpected inflammasome-activating capability of MPTP in mouse primary microglia. Similar to the effects observed in the mixed glial cells, MPTP/ATP stimulation triggered the robust secretion of active caspase-1 and mature IL-1β in WT mouse microglial cells (Fig. 3c,d). However, this MPTP/ATP-driven inflammasome activation was completely abrogated in *Nlrp3*^−/−^ microglia (Fig. 3c,d). Unlike the effects observed in the microglia, neither MPTP/ATP nor MPTP/LPS stimulation resulted in the activation of caspase-1 in mouse bone marrow-derived macrophages (BMDMs) (Supplementary Fig. 4b). As reported in the previous studies, MPTP can be converted into 1-methyl-4-phenyl-pyridinium (MPP^+^) by monoamine oxidase B, which is primarily expressed in glial cells [26]. In the MPTP-induced PD model, MPP^+^, rather than MPTP, is thought to exert neurotoxic effects via the generation of mitochondrial reactive oxygen species (ROS) inside dopaminergic neurons [26, 27]. Notably, MPP^+^ stimulation, when followed by treatment with ATP, induced the robust activation of caspase-1 in BMDMs (Fig. 3e). Conversely, neither MPP^+^ alone nor MPP^+^/LPS treatment failed to activate caspase-1 in BMDMs (Fig. 3e). The MPP^+^/ATP-triggered caspase-1 activation was completely abolished in *Nlrp3*^−/−^ BMDMs (Fig. 3f), demonstrating that MPP^+^ mediates the activation of the NLRP3 inflammasome in BMDMs.

### MPTP mediates the assembly of the NLRP3 inflammasome in microglia and mixed glial cells

**Fig. 4:**
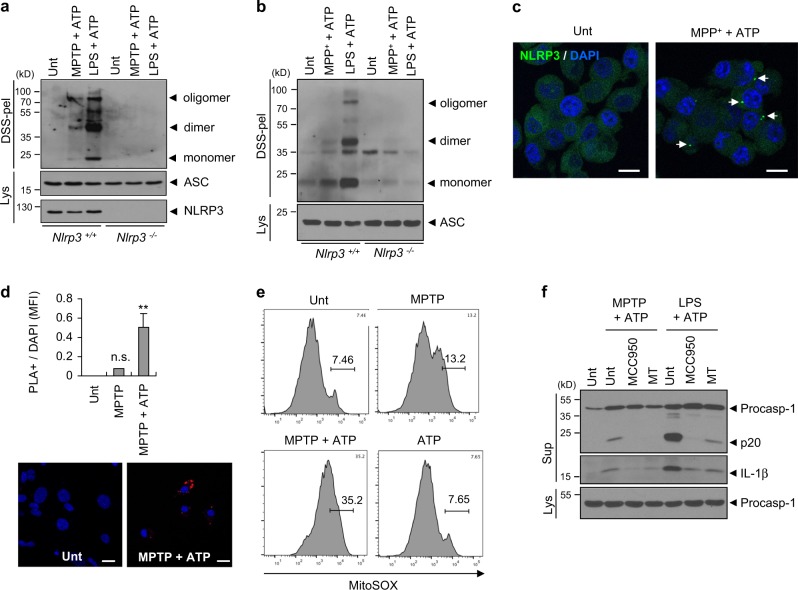
MPTP or MPP^+^ treatment promotes the assembly of the NLRP3 inflammasome. **a** Immunoblots of disuccinimidyl suberate (DSS)-crosslinked pellets (DSS-pel) or cellular lysates (Lys) from microglia treated with MPTP (40 μM, 16 h) or LPS (0.25 μg ml^−1^, 3 h), followed by the treatment of ATP (2 mM, 45 min). **b** Immunoblots of DSS-crosslinked pellets (DSS-pel) or cellular lysates (Lys) from BMDMs treated with MPP^+^ (100 μM, 6 h) or LPS (0.25 μg ml^−1^, 3 h), followed by the treatment of ATP (2 mM, 45 min). **c** Confocal images of NLRP3-GFP-expressing BMDMs untreated or treated with MPP^+^ (50 μM, 10 h) in the presence of ATP (2 mM, 30 min). Arrows indicate speck-like aggregates of NLRP3 protein (green). DAPI represents the nuclear signal (blue). Scale bars, 10 μm. **d** Proximity ligation assay of NLRP3 and ASC in mixed glial cells treated with MPTP (40 μM, 16 h), followed by the treatment of ATP (2 mM, 30 min). Proximity ligation (PL) signals (red) represent the molecular association of NLRP3 and ASC. Data are shown as a representative image from four or five independent samples (lower panel). Scale bars, 10 μm. The relative intensity of PL signals (per DAPI signals) was determined and is displayed in the upper panel (*n* = 4, 5). Data were expressed as the mean ± SEM. Asterisk indicates significant differences (**P < 0.01, n.s. not significant). **e** Flow cytometric analysis of mixed glial cells treated with MPTP (40 μM, 18 h) and ATP (2.5 mM, final 30 min) after staining with MitoSOX. **f** Immunoblots from mixed glial cells treated with MPTP (40 μM, 16 h) or LPS (0.25 μg/ml, 3 h) in the presence of MCC950 (50 nM) or Mito-TEMPO (MT, 200 μM), followed by ATP (2.5 mM, 30 min) treatment. **a**, **b**, **f** Culture supernatants (Sup), cellular lysates (Lys), or DSS-crosslinked pellets (DSS-pel) were analyzed by immunoblot

Next, we evaluated the oligomerization of ASC, an essential inflammasome adapter molecule, as an indication of the assembly of the NLRP3 inflammasome. Consequently, MPTP/ATP or MPP^+^/ATP stimulation induced the robust oligomerization of ASC in WT, but not in *Nlrp3*-deficient microglia or BMDMs (Fig. 4a,b). In accord with these observations, MPP^+^/ATP stimulation led to the formation of NLRP3 speck-like aggregates in NLRP3-GFP-expressing BMDMs, as observed by confocal microscopy (Fig. 4c). More intriguingly, MPTP/ATP treatment significantly increased the molecular interaction of NLRP3 and ASC in a mixed glial cell as determined by a proximity-based ligation assay (Fig. 4d), confirming that MPTP treatment mediates the activation of NLRP3, which, in turn, facilitates the association of NLRP3 with ASC.

To determine the mechanisms underlying the role of MPTP in the activation of the NLRP3 inflammasome, we first examined whether MPTP treatment could produce mitochondrial reactive oxygen species (ROS), considered as a potential mediator of NLRP3 inflammasome activation [28]. Previously report indicated that MPP^+^, which is derived from MPTP in glial cells, induces the generation of mitochondrial ROS via the inhibition of mitochondrial electron transport chain complex I [29]. Indeed, MPTP or MPTP/ATP treatment caused a marked increase in mitochondrial ROS production in mixed glial cells (Fig. 4e). Consistently, MPP^+^ treatment increased the MitoSOX-positive BMDM population (Supplementary Fig. 5a). Furthermore, MitoTEMPO, a mitochondrial ROS scavenger, clearly abrogated MPTP/ATP-triggered caspase-1 activation in a mixed glial culture (Fig. 4f) and microglia (Supplementary Fig. 5b). Collectively, these observations suggest that MPTP, and likely MPTP-derived MPP^+^, mediate the assembly and activation of the NLRP3 inflammasome via the production of mitochondrial ROS in microglia.

### MPTP functions as a priming signal for the NLRP3 inflammasome activation

**Fig. 5:**
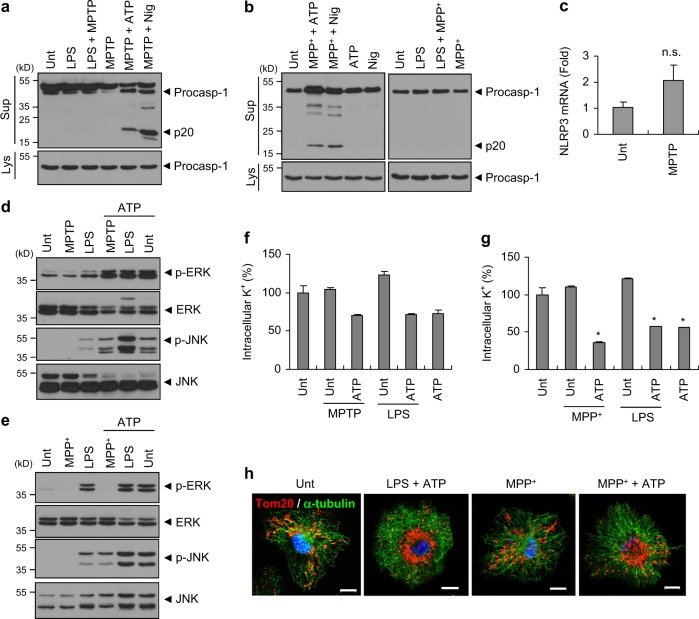
MPTP or MPP^+^ treatment alone does not induce the activation of mitogen-activated protein kinases and the efflux of potassium. **a** Immunoblots from mouse mixed glial cells untreated (Unt) or primed with LPS (0.25 μg ml^−1^, 3 h), followed by MPTP (40 μM, 14 h), or primed with MPTP, followed by ATP (2 mM, 40 min) or nigericin (5 μM, 45 min) treatment. **b** Immunoblots from mouse BMDMs untreated (Unt) or primed with MPP^+^ (100 μM, 6 h), followed by ATP (2.5 mM, 45 min) or nigericin (5 μM, 45 min, left panel), or primed with LPS (0.25 μg ml^−1^, 3 h), followed by MPP^+^ (100 μM, 6 h, right panel). **c** Cellular levels of NLRP3 mRNA in mouse mixed glial cells untreated or treated with MPTP (40 μM) for 18 h. (*n* = 7). **d**, **e** Immunoblots from mixed glial cells (**d**) or BMDMs (**e**) untreated or treated with MPTP (40 μM, 10 h, (**d**)), MPP^+^ (100 μM, 6 h, (**e**)) or LPS (0.25 μg ml^−1^, 3 h), followed by ATP treatment (2 mN, 40 min). **a**, **b**, **d**, **e** Culture supernatants (Sup) or cellular lysates (Lys) were immunoblotted with the indicated antibodies. **f**, **g** Intracellular K^+^ levels of mixed glial cells (**f**) or BMDMs (**g**) untreated or primed with MPTP (40 μM, 16 h, (**f**)), MPP^+^ (100 μM, 6 h, (**g**)) or LPS (0.25 μg ml^−1^, 3 h), followed by ATP (2 mM, 40 min) (*n* = 2, (**f**); *n* = 2, (**g**)). Asterisks indicate significant differences compared with untreated group (*P < 0.05). **h** Representative immunofluorescence images of mouse BMDMs untreated or treated with LPS (0.25 μg ml^−1^, 3 h), MPP^+^ (100 μM, 6 h), followed by ATP (2 mM, 40 min) treatment as stained with anti-Tom20 (red) and anti-α-tubulin (green) antibodies. DAPI represents the nuclear signal (blue). Scale bars, 10 μm

NLRP3 can be activated by various stimuli [30, 31]. Although the underlying mechanism of NLRP3 activation by diverse stimuli is still poorly understood, two-step activation mechanism by priming signal (signal 1) and activation signal (signal 2) is generally accepted [32]. We thus examined whether MPTP could function as a signal 1 or signal 2 for NLRP3 activation. As observed above, MPTP priming, followed by the treatment of ATP or nigericin, caused a robust caspase-1 activation in mixed glial cells, but MPTP treatment following LPS priming failed to induce caspase-1 activation (Fig. 5a). Likewise, MPP^+^ was able to activate caspase-1 only with post-treatment of ATP or nigericin, but not with LPS priming in BMDMs (Fig. 5b). These observations propose that MPTP or MPP^+^ is highly likely to act as a priming signal, rather than an activation signal, for NLRP3 activation. In this context, we tested whether MPTP treatment can upregulate the transcription of NLRP3 as a priming signal. Despite that MPTP triggers a remarkable induction of pro-IL-1β transcript levels in mixed glial cells (Fig. 3b), MPTP treatment did not significantly increase the expression level of NLRP3 mRNA (Fig. 5c).

In addition to the transcriptional effect of priming signals, recent studies also proposed that signal 1-induced post-translational modification of NLRP3 including deubiquitination or phosphorylation is a required priming step for the activation of NLRP3 [33, 34]. In particular, TLR-mediated activation of extracellular signal-regulated kinase (ERK) or c-Jun N-terminal kinase (JNK) pathway may contribute to the activation of NLRP3 inflammasome as an essential priming event [34, 35]. However, neither MPTP nor MPP^+^ alone induced the phosphorylation of ERK or JNK in mixed glial cells or BMDMs, whereas LPS treatment resulted in a robust phosphorylation of both proteins (Fig. 5d,e).

Next, we further assessed a potential effect of MPTP or MPP^+^ treatment on the signal 2-induced events such as potassium (K^+^) efflux and mitochondrial movement. As expected, ATP treatment alone was sufficient to drive K^+^ efflux in mixed glial cells or BMDMs (Fig. 5f,g). However, neither MPTP nor MPP^+^ stimulation significantly decreased intracellular K^+^ level in both cells (Fig. 5f,g).

Of interest, a recent study proposed that the signal 2-triggered mitochondrial movement into perinuclear regions plays a pivotal role in the NLRP3 activation [36]. In agreement with this previous study, our data suggest that LPS/ATP stimulation led to mitochondrial transport into perinuclear regions in BMDMs (Fig. 5h). This spatial mitochondrial rearrangement was also observed by MPP^+^/ATP treatment, but not by MPP^+^ alone (Fig. 5h). These findings demonstrate that MPP^+^ may function as a rather priming signal than activation signal for the activation of NLRP3 inflammasome.

### MPTP-mediated activation of the NLRP3 inflammasome in microglia causes neuronal cell death

**Fig. 6:**
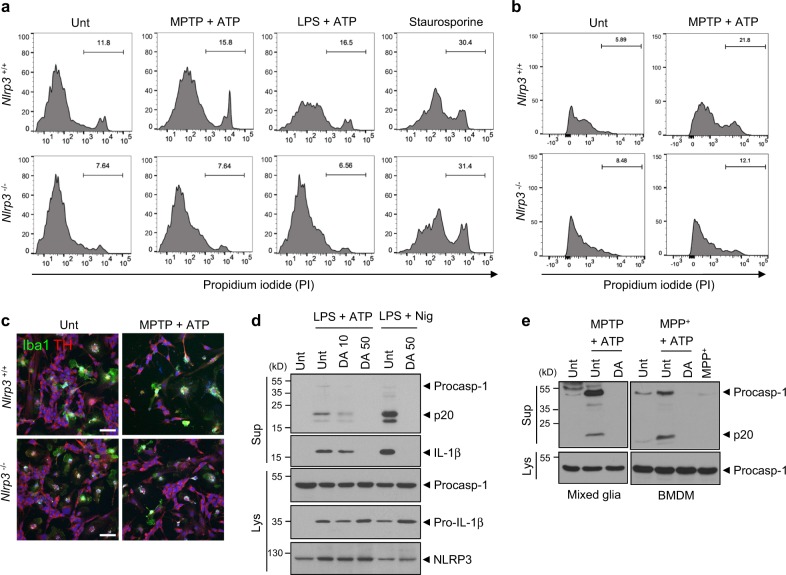
Microglial inflammasome activation mediates the death of dopaminergic neurons in a NLRP3-dependent manner. **a** Flow cytometric analysis of co-cultured microglia and SH-SY5Y cells treated with MPTP (40 μM, 16 h) or LPS (0.25 μg ml^−1^, 3 h), followed by the treatment with ATP (2.5 mM, 15 min), replenished with fresh medium and incubated for additional 24 h, or treated with staurosporine (1 μg ml^−1^, 16 h) after staining with anti-CD45 antibody and PI. The PI histograms of CD45-negative SH-SY5Y cells were displayed. **b** Flow cytometric analysis of co-cultured microglia and MN9D cells treated with MPTP (40 μM, 16 h), followed by the treatment with ATP (2.5 mM, 1 h) after staining with anti-CD45 antibody and PI. The PI histograms of CD45-negative MN9D cells were displayed. **c** Representative immunofluorescence images of co-cultured microglia and SH-SY5Y cells treated with MPTP (40 μM, 16 h), followed by treatment with ATP (2.5 mM, 15 min) after staining with anti-Iba1 (green) and anti-TH (red) antibodies. DAPI represents the nuclear signal (blue). Scale bars, 50 μm. **d** Immunoblots from mixed glial cells treated with LPS (0.25 μg ml^−1^, 3 h) in the presence of dopamine (10 or 50 μg ml^−1^, 30 min pretreatment before LPS), followed by ATP (2.5 mM, 30 min) or nigericin (Nig, 5 μM, 45 min) treatment. **e** Immunoblots from mixed glial cells (left) or BMDMs (right) treated with MPTP (40 μM, 16 h, left) or MPP^+^ (100 μM, 6 h, right) in the presence of dopamine (50 μg ml^−1^, 30 min pretreatment), followed by ATP (2 mM, 40 min) treatment. Culture supernatants (Sup) or cellular lysates (Lys) were immunoblotted with the indicated antibodies

To examine whether microglial NLRP3 inflammasome activation is responsible for the death of dopaminergic neurons, *Nlrp3*^+/+^ and *Nlrp3*^−/−^ microglia were co-cultured with the SH-SY5Y neuroblastoma cell line. After appropriate treatments, the co-cultured cells were gated on CD45 expression (Supplementary Fig. 6a). Cell death in CD45-negative SH-SY5Y cells was then determined by the cell-specific staining of dead cells with propidium iodide (PI). NLRP3-activating treatments, including MPTP/ATP and LPS/ATP, caused a marked increase in the population of PI-positive SH-SY5Y cells co-cultured with *Nlrp3*^+/+^ microglia but not in cells co-cultured with *Nlrp3*^−/−^ microglia (Fig. 6a). Given that these NLRP3-activating stimulations showed no direct cytotoxicity on SH-SY5Y cells (Supplementary Fig. 6b), microglia is probably responsible for the cell death of SH-SY5Y cells by inflammasome agonists. Furthermore, NLRP3-activating nigercin treatment in the presence of MPTP or LPS priming resulted in a significant increase in the PI-positive SH-SY5Y cells when co-cultured with microglia (Supplementary Fig. 6c). Conversely, the levels of staurosporine-induced apoptosis in SH-SY5Y cells were similar in the *Nlrp3*^+/+^ and *Nlrp3*^−/−^ microglial co-cultures (Fig. 6a). These results were further supported by similar experiments in a co-culture of microglia and MN9D cells, a murine dopaminergic cell line. The combined treatment with MPTP/ATP or LPS/ATP led to marked cell death in MN9D cells co-cultured with *Nlrp3*-expressing WT microglia, but not in cells co-cultured with *Nlrp3*-deficient microglia (Fig. 6b and Supplementary Fig. 7a).

To further confirm the neuronal loss via the activation of the microglial NLRP3 inflammasome, we observed co-cultures of microglia and SH-SY5Ycells using confocal microscopy. Consequently, we found that MPTP/ATP treatment clearly reduced the number of TH-positive SH-SY5Y cells following co-culture with *Nlrp3*^+/+^ microglia, but not with *Nlrp3*^−/−^ microglia (Fig. 6c and Supplementary Fig. 7b).

The loss of dopaminergic neurons led to a subsequent decrease in dopamine levels in PD patients [37]. Interestingly, dopamine was previously shown to suppress the activation of the NLRP3 inflammasome in BMDMs [38]. Consistent with this previous study, dopamine treatment markedly diminished the activation of the NLRP3-dependent inflammasome in mixed glial cells stimulated with NLRP3 agonists (Fig. 6d) and with MPTP/ATP treatment (Fig. 6e). These findings further suggest that reduced dopamine levels could exacerbate neuroinflammation in the central nervous system by hindering the inflammasome-suppressing capability of dopamine.

### Administration of IL-1 receptor antagonist attenuates MPTP-induced PD phenotypes in mice

**Fig. 7:**
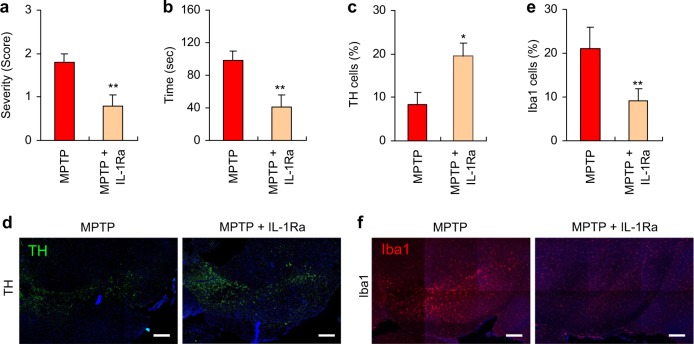
Administration of interleukin-1 receptor antagonist attenuates motor dysfunctions and dopaminergic neuronal loss in MPTP-treated mice. **a** The severity of hindlimb clasping in MPTP-treated mice with or without IL-1Ra administration (*n* = 10). **b** The latent time to hold the bar in the catalepsy test in MPTP-treated mice with or without IL-1Ra administration (*n* = 10). **c** Quantification of relative TH-positive cells per DAPI in the confocal images as shown in (**d**) (*n* = 5). **e** Quantification of relative Iba1-positive cells in the confocal images as shown in **f** based on DAPI staining (*n* = 4 or 5). **d**, **f** Representative immunofluorescence image of the fixed brain sections containing the SN of MPTP-treated mice with or without IL-1Ra administration after staining with anti-TH antibody (green, **d**) or anti-Iba1 antibody (red, **f**). DAPI represents the nuclear signal (blue). Scale bars, 200 μm. Data were expressed as the mean ± SEM. Asterisks indicate significant differences (*P < 0.05, **P < 0.01)

To confirm if microglial NLRP3 inflammasome activation plays a key role in our MPTP-induced PD mouse model, we tested the effect of an IL-1 receptor antagonist (IL-1Ra), which prevents inflammasome-driven IL-1β signaling by blocking the IL-1 receptor. The administration of IL-1Ra significantly reduced the motor deficits in MPTP-treated mice as determined by the severity of hindlimb clasping (Fig. 7a) and the cataleptic response (Fig. 7b). We likewise examined the potential effect of IL-1Ra on the MPTP-induced loss of dopaminergic neurons, and found that TH-positive dopaminergic neurons were significantly increased in the SN of MPTP-treated mice following the administration of IL-1Ra (Fig. 7c, d). In accord with these findings, IL-1Ra markedly attenuated the MPTP-induced increase in the microglial population in the SN (Fig. 7e,f). These data demonstrate that the inflammasome-dependent production of IL-1β plays a crucial role in the progression of the PD phenotype in MPTP-treated mice.

### Conditional microglia-specific expression of a constitutively active NLRP3 mutant exacerbates MPTP-induced PD phenotypes in mice

**Fig. 8:**
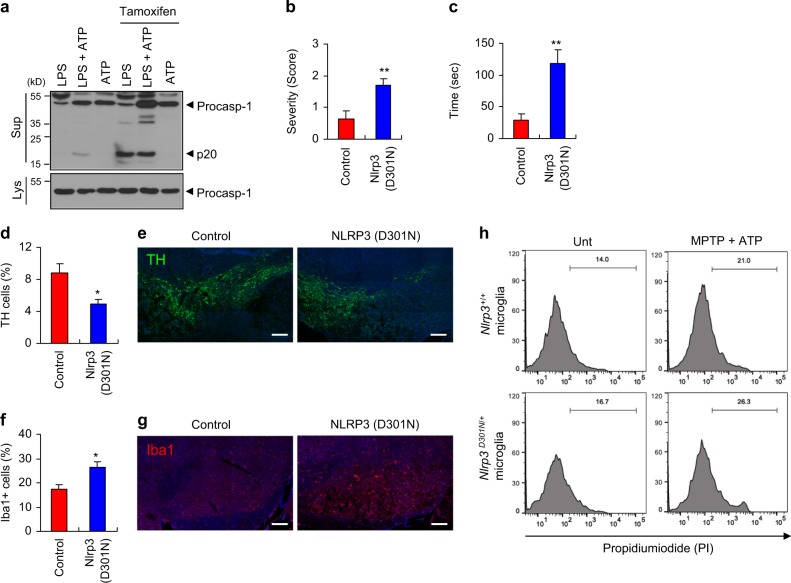
Conditional expression of NLRP3 active mutant in microglia exacerbates motor dysfunctions and dopaminergic neuronal loss in MPTP-treated mice. **a** Immunoblots from *Cx3Cr1*^*Cre-ER/+*^*Nlrp3*^*D301NneoR/+*^ mouse mixed glial cells treated with tamoxifen (1 μM, 24 h), replenished with the medium containing LPS (0.25 μg ml^−1^, 3 h), followed by ATP (2 mM, 40 min). **b**–**g** Control (*Cx3Cr1*^*Cre-ER/Cre-ER*^*Nlrp3*^*+/+*^) or NLRP3 mutant mice (*Cx3Cr1*^*Cre-ER/+*^*Nlrp3*^*D301NneoR/+*^) were treated with tamoxifen for 5 days, followed by the administration of MPTP for additional 3 days. **b** The severity of hindlimb clasping in MPTP-treated control or *Nlrp3* (D301N)-expressing mice. (control, *n* = 8; *Nlrp3* (D301N), *n* = 7). **c** The latent time to hold the bar in the catalepsy test in MPTP-treated control or *Nlrp3* (D301N)-expressing mice (control, *n* = 8; *Nlrp3* (D301N), *n* = 7). **d** Quantification of relative TH-positive cells per DAPI in the confocal images as shown in (**e**) (control, *n* = 5; *Nlrp3* (D301N), *n* = 3). **f** Quantification of relative Iba1-positive cells per DAPI in the confocal images as shown in (**g**) (control, *n* = 5; *Nlrp3* (D301N), *n* = 3). **e**, **g** Representative immunofluorescence image of the fixed brain sections containing the SN of MPTP-treated control or *Nlrp3* (D301N)-expressing mice following tamoxifen treatment after staining with anti-TH antibody (green, **e**) or anti-Iba1 antibody (red, **g**). DAPI represents the nuclear signal (blue). Scale bars, 200 μm. **h** *Cx3Cr1*^*Cre-ER/Cre-ER*^*Nlrp3*^*+/+*^ or *Cx3Cr1*^*Cre-ER/+*^*Nlrp3*^*D301NneoR/+*^ mice microglia was treated with tamoxifen (1 μM, 24 h), and co-cultured with SH-SY5Y cells. Flow cytometric analysis of co-cultured *Nlrp3*^*+/+*^ or *Nlrp3*^*D301N/+*^ microglia and SH-SY5Y cells treated with MPTP (40 μM, 16 h), followed by the treatment with ATP (2.5 mM, 15 min), replenished with fresh medium and incubated for additional 24 h, after staining with anti-CD45 antibody and PI. The PI histograms of CD45-negative SH-SY5Y cells were displayed. Data were expressed as the mean ± SEM. Asterisks indicate significant differences (*P < 0.05, **P < 0.01)

Mutations in *NLRP3* are frequently found in patients with autoinflammatory diseases, including neonatal-onset multisystem inflammatory disease and Muckle-Wells syndrome [39]. NLRP3 mutant D301N, which corresponds to human D303N mutation identified in the patients of autoinflammatory diseases, is considered as a constitutively active form of NLRP3 [40]. Furthermore, the knock-in mice expressing NLRP3 (D301N) exhibited severe systemic inflammatory phenotypes [41, 42]. To verify that microglial NLRP3 inflammasome signaling contributes to the observed MPTP-induced PD symptoms, we generated knock-in mice harboring a conditional *Nlrp3* (D301N) allele (floxed) and the CX_3_CR1-Cre-estrogen receptor (ER). Following the administration of tamoxifen, the Cre recombinase is activated and the NLRP3 (D301N) mutant is subsequently expressed exclusively in the microglia under the control of CX_3_CR1. Of notice, tamoxifen-induced expression of NLRP3 mutant in microglia greatly enhanced caspase-1 activation in response to LPS stimulation (Fig. 8a). Then, we examined the effect of CX_3_CR1-specific NLRP3 mutant expression on the MPTP-induced motor deficits in mice. After the administration of MPTP for only 3 days, *Nlrp3* (D301N)-expressing mice exhibited profound motor dysfunctions, as determined by hindlimb clasping (Fig. 8b) and catalepsy tests (Fig. 8c). In addition, a severe truncal dystonia was observed in these NLRP3 mutant mice compared with *Nlrp3* ^+/+^ control mice (Supplementary Fig. 8a). Furthermore, this conditional expression of *Nlrp3* mutant in microglia led to a significant reduction in TH-positive dopaminergic neuron in the SN of mice following only 3 days of MPTP treatment (Fig. 8d,e). To support these observations, MPTP-treated *Nlrp3* (D301N)-expressing mice showed a significantly increased the microglial population in the SN (Fig. 8f,g). Consistently, tamoxifen-induced expression of *Nlrp3* mutant in microglia exhibited an increased cytotoxicity to co-cultured SH-SY5Y cells (Fig. 8h). Furthermore, active IL-1β was clearly detected only in the SN of *Nlrp3* mutant-expressing mice upon 3 days of MPTP administration (Supplementary Fig. 8b). These additional findings clearly support that the NLRP3 inflammasome activation in microglia plays a central role in MPTP-induced PD pathogenesis.

## Discussion

Recently, inflammasome activation has been implicated in the progression of neurodegenerative disorders such as Alzheimer's disease and multiple sclerosis [17, 43, 44]. Interestingly, the loss of dopaminergic neurons in the SN was significantly inhibited by the genetic ablation of caspase-1 or by treatment with a selective caspase-1 inhibitor in an animal PD model following the administration of MPTP or 6-hydroxydopamine administration [20, 45]. Furthermore, Yan et al. demonstrated that dopaminergic neuronal loss was attenuated by NLRP3 deficiency in the MPTP-treated mouse model [38]. These previous reports implicated the inflammasome signaling pathways in PD pathogenesis, but the mechanisms underlying the contribution of the caspase-1/inflammasome pathways to the neurodegeneration observed in PD remain unclear. Accordingly, we attempted to examine the mechanistic basis of the observed inflammasome-mediated neurodegeneration.

A recent study reported that active caspase-1 cleaves α-synuclein into an aggregation-prone form, suggesting that inflammasome signaling potentially contributes to the formation of α-synuclein aggregates [21]. On the other hand, Qiao et al. demonstrated that the neuronal activation of caspase-1 by MPP^+^ causes cell death in SH-SY5Y cells via caspase-7-dependent apoptosis [20]. These recent studies suggest a potential neurodegenerative role of inflammasome signaling inside dopaminergic neurons, but the expression of inflammasome components, such as NLRP3 or ASC, in dopaminergic neurons might be very low and required further clarification. Instead, our results here present evidence that MPTP-driven NLRP3 activation in microglia is directly responsible for neuronal death. Notably, our data indicate that the microglia-specific conditional expression of a constitutively active NLRP3 mutant greatly exacerbated the MPTP-induced motor dysfunctions and loss of dopaminergic neurons in mice. These results indicate that NLRP3 inflammasome-active microglia play a critical role in MPTP-induced neurodegeneration.

At present, the downstream target of the NLRP3 inflammasome that causes cell death in dopaminergic neurons is not completely understood. Based on the observation that IL-1Ra attenuates MPTP-induced motor dysfunctions and dopaminergic neuronal loss, IL-1β signaling might contribute to the recruitment of microglia and the related neurotoxicity in vivo. Nonetheless, based on the microglia-neuron co-culture results, the activation of the NLRP3 inflammasome appears to increase the neurotoxic potential of microglia. Therefore, further studies are required to determine whether microglia trigger the secretion of neurotoxic compounds in a NLRP3 inflammasome-dependent manner. Of particular interest, a recent study demonstrates that microglia-derived ASC specks bind to amyloid-β and induce the formation of amyloid-β aggregates in Alzheimer's disease [25]. Similarly, we also found the formation of ASC specks in the substantia nigra of NLRP3-expressing MPTP-treated mice. Therefore, it will be interesting to investigate whether NLRP3-dependent ASC specks are able to cross-seed α-synuclein in PD pathology.

In the present study, we found that MPTP, a compound commonly used to induce PD in animal studies [27], triggered the assembly and activation of the NLRP3 inflammasome in microglia. Considering that many endogenous or exogenous toxic substances are able to activate NLRP3 [46, 47], our data indicated that MPTP is a novel exogenous stimulator for the NLRP3 inflammasome in microglia possibly via MPP^+^-mediated production of mitochondrial ROS. Collectively, our results indicate that the NLRP3 inflammasome plays a crucial role in the neurotoxic effects of microglia and the subsequent loss of dopaminergic neurons in MPTP-treated mice and cell culture models. Therefore, our data suggest that microglial NLRP3 inflammasome signaling might serve as a therapeutic target for preventing the progression of PD.

## Materials and methods

### Mice

C57BL/6, *Nlrp3*^−/−^, and *Cx*_*3*_*Cr1*^*CreER*^ (C57BL/6 background) mice were obtained from The Jackson Laboratory and bred at Yonsei University College of Medicine. All of the mice were maintained under specific pathogen-free conditions and 9–12 weeks male mice were used for the experiments. Protocols for the animal experiments were approved by the Institutional Ethical Committee, Yonsei University College of Medicine. All experiments were performed in accordance with the approved guidelines of the Institutional Ethical Committee.

### Mice treatment

The administration of MPTP was used to induce PD-like phenotype in mice according to previous study with slight modifications [24]. In brief, the mice were administered an intraperitoneal injection of 30 or 40 mg kg^−1^ MPTP for 5 days at 24 h intervals. In some experiments, the mice were also given 5 mg kg^−1^ of an IL-1 receptor antagonist (Prospec) intraperitoneally on the 1st and 3rd day during the administration of the MPTP injections.

### Conditional expression of the NLRP3 mutant in mouse brain microglia

To induce the microglia-specific expression of the NLRP3 (D301N) mutant, *Nlrp3*^*D301NneoR*^ mice harboring a loxP-flanked neomycin resistant cassette (reverse orientation) in intron 2 and a point mutation in exon 3 of *Nlrp3* were bred to *Cx*_*3*_*Cr1*^*Cre-ER*^ mice. Pups (12 weeks old) were treated with tamoxifen (75 mg/kg) for 5 days to induce the expression of active Cre recombinase, resulting in the excision of the neomycin resistant cassette and the expression of NLRP3 (D301N) in Cx3Cr1-specific cells (*Nlrp3*^*D301N/+*^), including the microglia.

### Behavioral tests

For all of the tests, the mice were transported to the experimental room and allowed to adjust to the environment for 30 min. Behavioral tests were performed 1 h or 24 h after the final PBS or MPTP injection. To measure forelimb strength and coordination, a string test was used as previously described [48]. Briefly, a narrow horizontal bar was fixed between two vertical supports 20 cm above the floor, and the mice were hung on the central point of the bar by the forepaws for 3 min, and the holding time was then measured. Catalepsy was measured as previously described [49]. A horizontal bar (3 mm diameter) was placed 5 cm above the floor, and the mice were hung on the bar with their forepaws and hindpaws on the floor. The time to move the forepaws off the bar was measured for 3 min. To measure the hindlimb clasping, the mice were suspended by the tail, and hindlimb clasping was scored from 0 to 3 based on the following scale as previously described: [50] 0, hindlimbs were fully spread out away from the abdomen; 1, one hindlimb was closed toward the abdomen; 2, both hindlimbs partially closed toward abdomen; 3, both hindlimbs were completely closed toward the abdomen.

### Reagents and antibodies

MPTP was obtained from Sigma-Aldrich or Toronto Research Chemicals. MPP^+^, LPS, ATP, nigericin, staurosporine, propodium iodide, MCC950, dopamine and glibenclamide were purchased from Sigma-Aldrich. Mito-TEMPO was obtained from ENZO Life Sciences. MitoSOX was purchased from Invitrogen. Anti-mouse caspase-1 (AG-20B-0042) and anti-NLRP3 (AG-20B-0014) antibodies were obtained from Adipogen. Anti-mouse IL-1β antibody was obtained from R&D Systems (AF-401-NA). Anti-ASC antibody (SC-22514) and anti-phospho-ERK antibody (SC-7383) were purchased from Santa Cruz. Anti-CD45-BV421 antibody was obtained from BD Biosciences. Anti-ERK antibody (4695) and anti-phospho-JNK antibody (9251) were purchased from Cell Signaling. Anti-JNK antibody (554285) was obtained from BD pharmingen.

### Immunohistochemistry

Mice were sacrificed 25 h after the final administration of PBS or MPTP, and perfused with PBS and 4% paraformaldehyde. For the frozen sections, the brains were fixed with 4% paraformaldehyde, and dehydrated with 30% sucrose solution until the brain sunk to the bottom at 4 °C. OCT-embedded frozen blocks were cut by Leica CM1860 cryostat (20 μm thick). The sections were then stained with primary antibodies targeting tyrosine hydroxylase (TH, Millipore or R&D), Iba-1 (Wako or Novus) or ASC (Santa Cruz), followed by treatment with Cy3-, FITC-, or Alexa 488-labeled secondary antibodies. DAPI counterstaining was used to visualize the nuclei. Images of the cell were acquired and processed with confocal microscopy (LSM 700, Carl Zeiss) and the ZEN2011 software. TH and Iba-1-positive cells in the selected regions were counted using the Image J software. For Nissl staining, the brain sections were stained with cresyl violet using a standard protocol.

### Determination of dopamine levels

To determine the striatal dopamine level of mouse brain, dissected striatum of mouse brain was homogenized in RIPA buffer (50 mM Tris, pH 8.0, 150 mM NaCl, 1% Nonidet P-40, 0.5% sodium deoxycholate and protease inhibitors. Homogenates were normalized by Bradford assay, and the dopamine levels of each sample were quantified by high performance liquid chromatography-tandem mass spectrometry (HPLC-MS/MS). In order to extract the target substances while removing the detergent from the sample, liquid–liquid extraction using water-saturated ethyl acetate was applied. The separation was obtained on Phenomenex Kinetex C18 column and detection of the ions was conducted in selective reaction monitoring mode. Dopamine *m/z* 154 > 137 and d4-DA (internal standard) *m/z* 158 > 141.

### Cell cultures

Mouse BMDMs were prepared from C57BL/6 or *Nlrp3*^−/−^ mice as previously described [51]. Immortalized NLRP3-GFP-expressing BMDMs were provided by Dr. E.S. Alnemri (Thomas Jefferson University, Philadelphia, USA). All BMDMs were maintained in L929-conditioned DMEM supplemented with 10% FBS and antibiotics. Mouse brain mixed glial cells were prepared from the whole brains of mouse pups on postnatal day 1–3 and cultured for 3 weeks as previously described [52]. Microglial cells were further enriched from the mixed glial cultures using mild trypsinization as previously described [53]. The microglia cultures were co-stained with anti-CD11b and anti-F4/80 antibodies (eBioscience) and characterized by flow cytometry. Mixed glial cells and microglia were grown in DMEM/F12 (1:1) supplemented with 10% FBS and antibiotics. SH-SY5Y cells were maintained with DMEM/F12 supplemented with 10% FBS and antibiotics. MN9D cells were kindly provided by Dr. Y.J. Oh (Yonsei University, Seoul, Korea), and cultured on poly-D-lysine-coated plates in DMEM.

### Immunoblot analysis

Cells were lysed in buffer containing 20 mM HEPES (pH 7.5), 0.5% Nonidet P-40, 50 mM KCl, 150 mM NaCl, 1.5 mM MgCl_2_, 1 mM EGTA, and protease inhibitors. Soluble lysates were fractionated by SDS-PAGE and then transferred to PVDF membranes. In some experiments, cell culture supernatants were precipitated by methanol/chloroform as described previously [54] and then immunoblotted. All the blots shown are representative images of at least three independent experiments.

### Quantification of mRNA

To measure mRNA production, total RNA was isolated using RNeasy Mini Kit (Intron) or TRIzol reagent (Invitrogen) and reverse transcribed using Power cDNA Synthesis Kit (Intron). Quantitative real-time PCR was performed using SYBR Premix Ex Taq (Takara). Primers were as follows: 5′-GCC CAT CCT CTG TGA CTC AT-3′ and 5′-AGG CCA CAG GTA TTT TGT CG-3′ (mouse *Il-1β*); 5′-AGT TGC CTT CTT GGG ACT GA-3′ and 5′-TCC ACG ATT TCC CAG AGA AC-3′ (mouse *Il-6*); 5′- ATG CTG CTTCGA CAT CTC CT-3′ and 5′-AAC CAA TGC GAG ATC CTG AC-3′ (mouse *Nlrp3*); 5′-CGC GGT TCT ATT TTG TTG GT-3′ and 5′-AGT CGG CAT CGT TTA TGG TC-3′ (mouse *Rn18s*).

### Assay of inflammasome/caspase-1 activation

To induce a conventional NLRP3 inflammasome activation, BMDMs or microglial cells were primed with LPS (0.25 μg ml^−1^, 3 h), followed with ATP treatment (2–2.5 mM, 30–45 min). Inflammasome activation was determined by the presence of active caspase-1 p20 and active IL-1β from culture supernatants in immunoblots, and by the extracellular IL-1β quantification using ELISA. FAM-FLICA^TM^-caspase-1 assay kit (ImmunoChemistry) was employed to detect active caspase-1 in the brain sections according to the manufacturer's protocol.

### Assay of NLRP3 inflammasome assembly

To measure the oligomerization of NLRP3, speck-like aggregates of NLRP3-GFP were assessed by confocal microscopy in NLRP3-GFP-expressing BMDMs. To determine the oligomerization of ASC, discuccinimidyl suberate (DSS, Thermo Scientific)-mediated cross-linking assay was performed as described previously [55]. To visualize the molecular interaction of NLRP3 with ASC, proximity-ligation assay was performed using Duolink In Situ Red starter kit (Sigma) using anti-ASC or anti-NLRP3 antibodies according to the manufacturer's protocols. The relative proximity ligation signals (PL signals/DAPI signals) were quantified using the Image J software and calculated as a relative fold-change compared to untreated controls.

### Assay of mitochondrial ROS production

To measure mitochondrial ROS production levels, mixed glial cells or mouse BMDMs were stained with MitoSOX (Invitrogen) after the appropriate treatments. Cells were then analyzed by flow cytometry based on the level of MitoSOX (FACSVerse, BD).

### Determination of intracellular K^+^ levels

Intracellular K^+^ concentration was measured by inductively coupled plasma-optical emission spectrometry (ICP-OES) using an OPTIMA 8300 ICP spectrometer (Perkin Elmer) as described previously [56]. Cells were extracted with 10% HNO3 and intracellular K^+^ levels were determined.

### Flow cytometry to determine neuronal cell death

To measure microglia-induced neuronal degeneration, microglia from *Nlrp3*^+/+^ or *Nlrp3*^−/−^ mice were co-cultured with neuroblastoma SH-SY5Y cells or dopaminergic MN9D cells. Following appropriate treatments, neuronal cell death in the co-cultures was determined by flow cytometry or immunofluorescence assay. For the flow cytometry analyses, cells were stained with anti-CD45 antibody and propidium iodide (PI). Cells were gated on CD45 expression, and the CD45-negative neurons were analyzed with the fluorescence of PI by flow cytometry.

### Immunofluorescence assay

Cells grown on coverslip in a 12-well plate were fixed with 4% formaldehyde and permeabilized with 0.2% Triton X-100. After blocking with 4% BSA, cells were incubated with primary antibodies targeting tyrosine hydroxylase (TH, Millipore), Iba-1 (Wako), Tom 20 (Santa Cruz) or α-tubulin (Santa Cruz), followed by the Cy3-, Alexa Fluor 488-, or FITC-conjugated anti-mouse or anti-rabbit IgG (Jackson Immuno Research or Invitrogen). Cells were then observed by confocal microscopy (Zeiss, LSM700).

### Statistical analysis

All values were expressed as the mean ± SEM of individual samples. Data were analyzed using one-way analysis of variance (ANOVA) followed by Dunnett's post hoc test for comparison of all groups with control group, two-way ANOVA with Bonferroni post hoc test for comparison between WT and *Nlrp3*-deficient mouse groups, or Student's *t* tests. The level of statistical significance was set at *P* ≤ 0.05. Analyses were performed using GraphPad Prism.

## Electronic supplementary material

Supplementary Figure

Supplementary discussion
