# Supplementary material for: MPTP-driven NLRP3 inflammasome activation in microglia plays a central role in dopaminergic neurodegeneration
Source: Cell Death Differ. 2018 May 21;26(2):213–28. doi: 10.1038/s41418-018-0124-5 (PMC6329843; doi:10.1038/s41418-018-0124-5)
Supplement: Supplementary file 1 — Supplementary Figure [file 41418_2018_124_MOESM1_ESM.pdf]

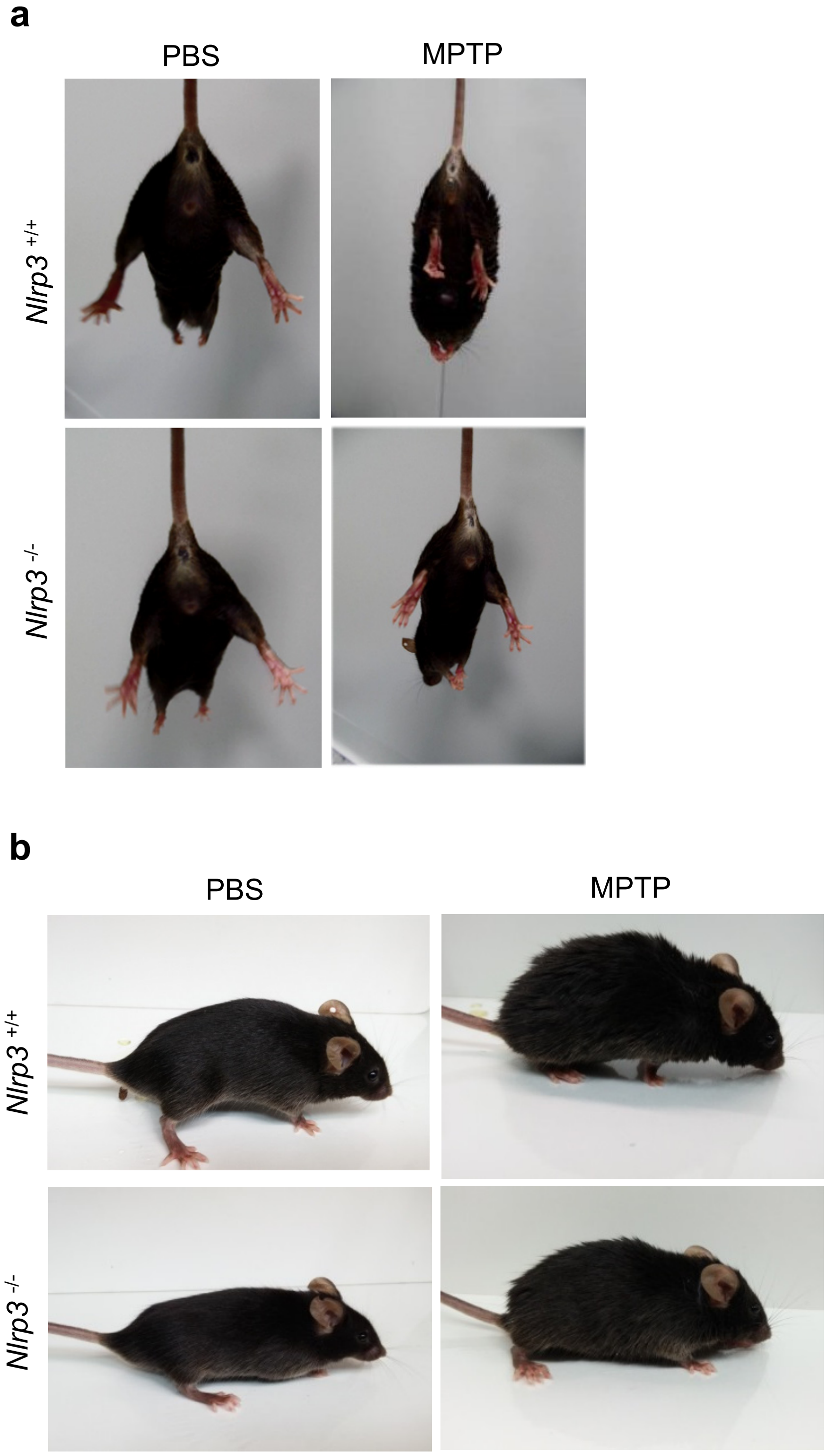

Supplementary Figure 1. MPTP-induced PD phenotypes of wild-type and *Nlrp3* $^{-/-}$ mice. (**a**) Hindlimb clasping test. (**b**) Truncal dystonia.

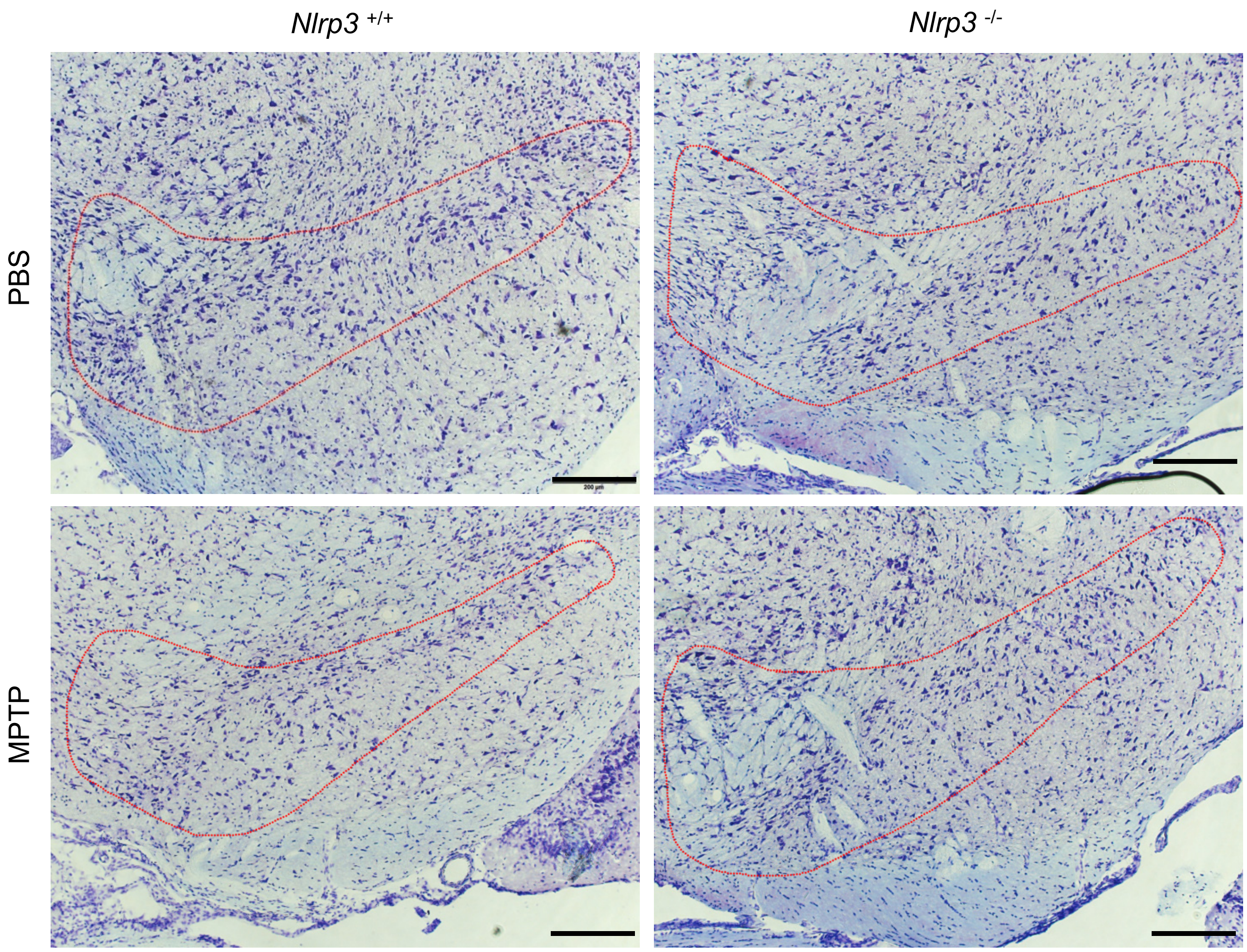

Supplementary Figure 2. Nissl staining of substantial nigra regions of PBS- or MPTP-treated mice. Representative Nissl-stained image of the fixed brain sections containing SN of PBS- or MPTP-treated *Nlrp3* $^{+/+}$ or *Nlrp3* $^{-/-}$ mice after staining with cresyl violet. Substantia nigra pars compacta is outlined with a red dashed line. Scale bars, 200 μm.

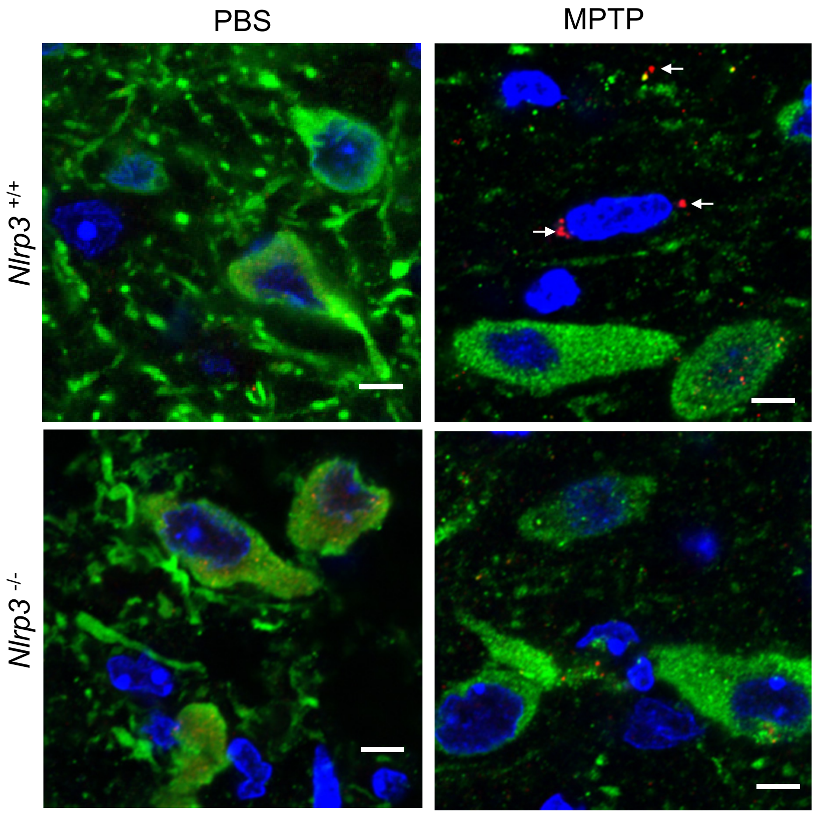

Supplementary Figure 3. ASC speck formation in the substantia nigra sections of MPTP-treated mice. Representative immunofluorescence images of substantia nigra pars reticulata sections of PBS- or MPTP-treated *Nlrp3* $^{+/+}$ or *Nlrp3* $^{-/-}$ mice after staining with anti-TH (green) and anti-ASC (red) antibodies. DAPI represents the nuclear signal (blue). Arrows indicate the speck-like ASC aggregates. Scale bars, 10 μm.

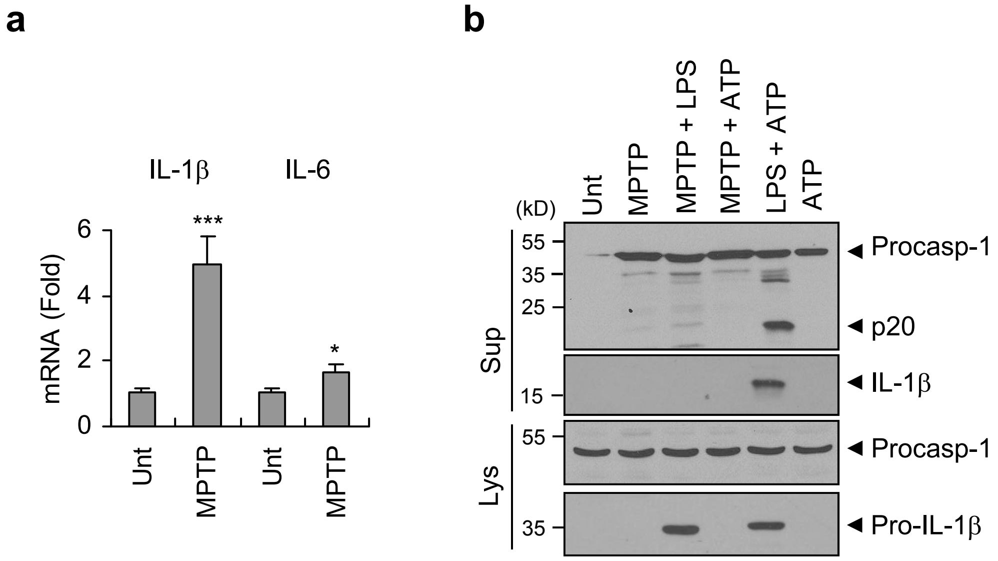

Supplementary Figure 4. (**a**) Mouse mixed glial cells were untreated (Unt) or treated with MPTP (40 μM) for 18 h. The IL-1β or IL-6 mRNA levels in the cell extracts were then assayed using quantitative real-time PCR (n = 7). (**b**) Mouse BMDMs were pretreated with MPTP (40 μM) for 16 h, followed by treatment with LPS (0.25 μg/ml, 3 h), ATP (2.5 mM, 30 min), or were pretreated with LPS, followed by ATP treatment as indicated. Culture supernatants (Sup) or cellular lysates (Lys) were immunoblotted with the indicated antibodies. Unt, untreated.

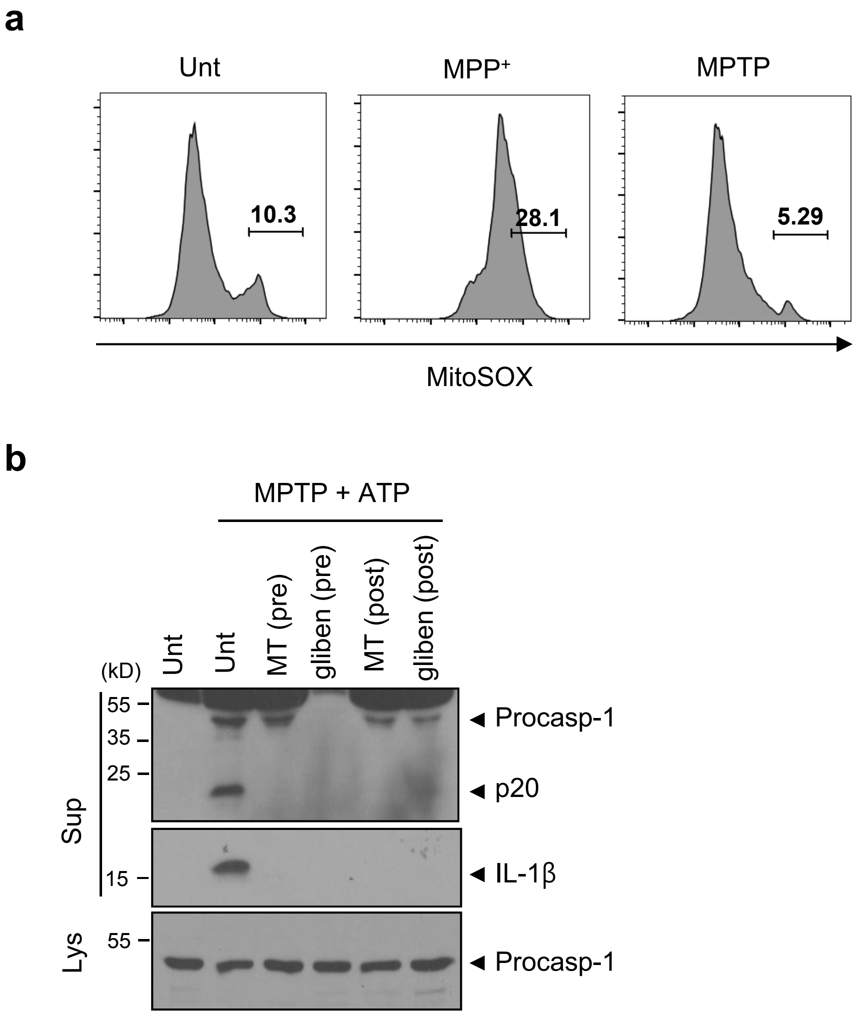

Supplementary Figure 5. (**a**) Mouse BMDMs were treated with MPP^+ (40 μM) or MPTP (40 μM) for 18 h, and the mitochondrial ROS levels were assayed after MitoSox staining. (**b**) Mouse microglia were pretreated with MPTP (40 μM) for 18 h, followed by treatment with ATP (2.5 mM) for 30 min. MitoTEMPO (MT, 200 μM) or glibenclamide (50 μM) were administered together with MPTP (pre) or 30 min before ATP (post). Culture supernatants (Sup) or cellular lysates (Lys) were immunoblotted with the indicated antibodies. Unt, untreated.

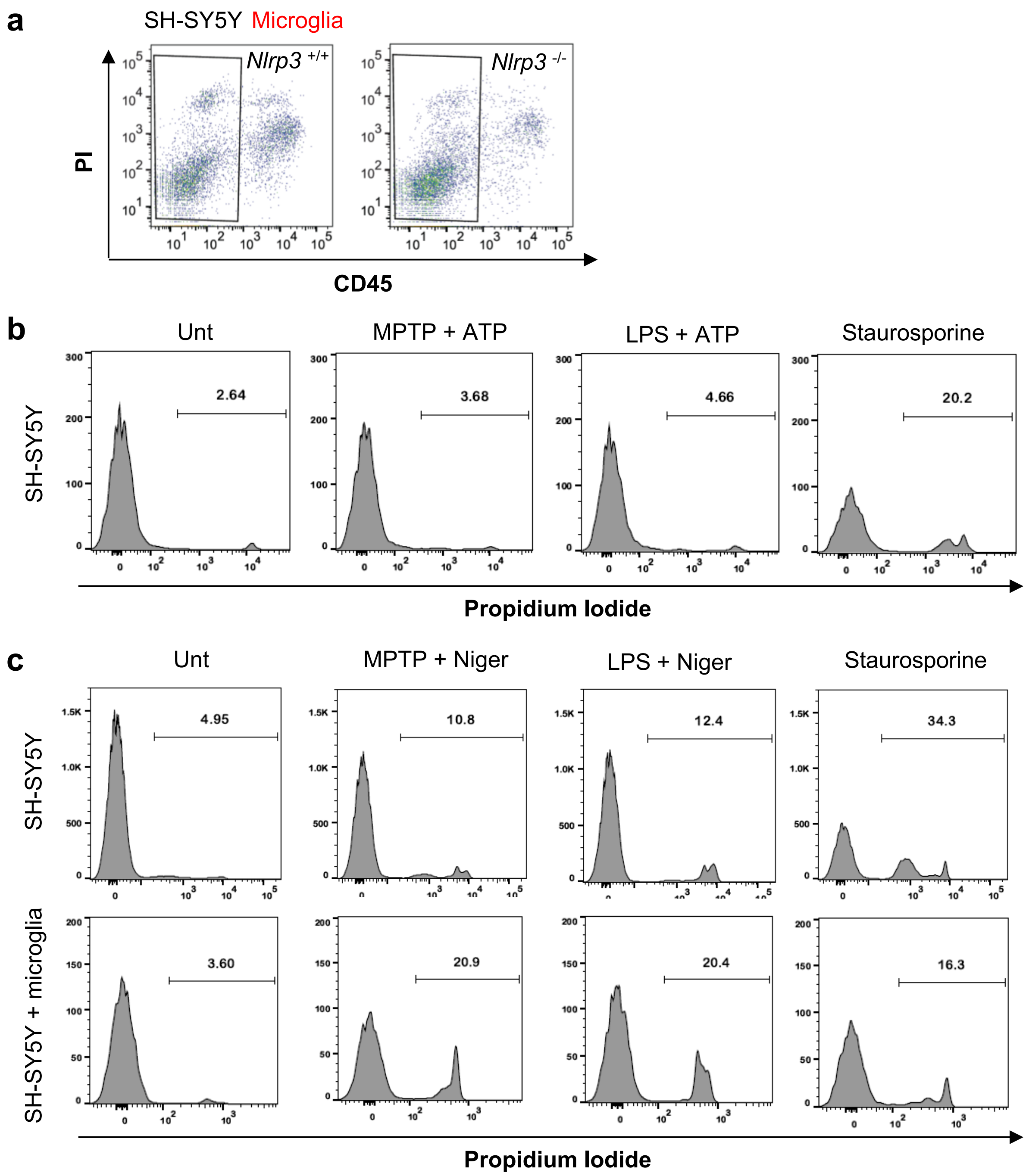

Supplementary Figure 6. (**a**) Microglia, prepared from *Nlrp3*$^{+/+}$ or *Nlrp3*$^{-/-}$ mice, were co-cultured with SH-SY5Y cells. The co-cultured cells were stained with anti-CD45 antibody and propidium iodide (PI), and then analyzed by flow cytometry. (**b**) SH-SY5Y cells were treated with MPTP (40 μM, 16 h) or LPS (0.25 μg/ml, 3 h), followed by treatment with ATP (2.5 mM, 20 min), then replenished with fresh medium and incubated for additional 24 h, or cells were treated with staurosporine (1 μg/ml, final 16 h). Then, cells were stained with PI and analyzed by flow cytometry. (**c**) SH-SY5Y cells alone or together with *Nlrp3*$^{+/+}$ microglia were treated with MPTP (50 μM, 18 h) or LPS (0.25 μg/ml, 3 h), followed by treatment with nigericin (5 μM, 30 min), then replenished with fresh medium and incubated for additional 24 h, or treated with staurosporine (1 μg/ml, final 18 h). Cells were then stained with anti-CD45 and the PI histograms of CD45-negative SH-SY5Y cells were displayed.

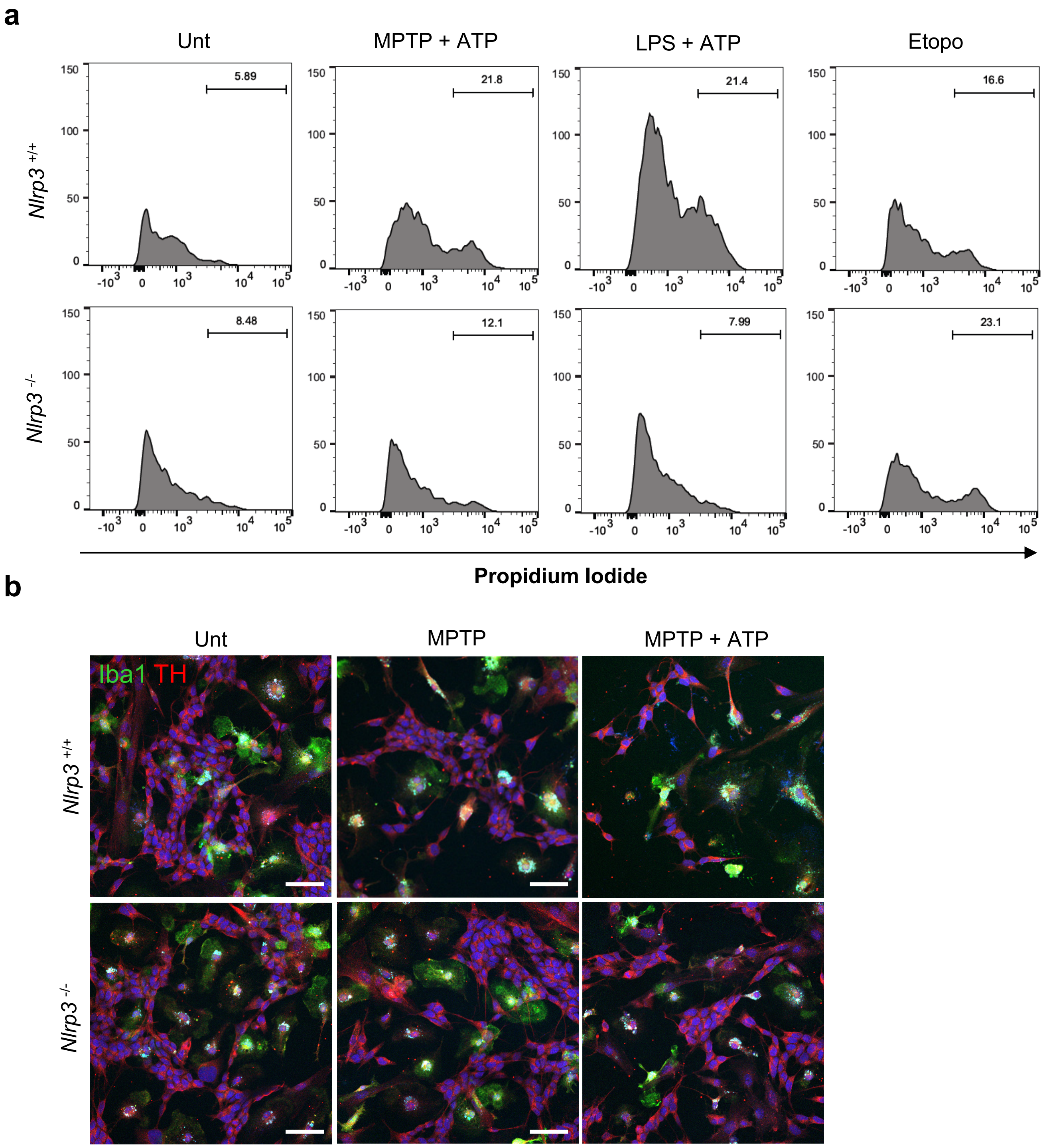

Supplementary Figure 7. (**a**) *Nlrp3*$^{+/+}$ or *Nlrp3*$^{-/-}$ microglia were co-cultured with MN9D cells. The co-cultured cells were pretreated with MPTP (40 μM, 16 h) or LPS (0.25 μg/ml, 3 h), followed by treatment with ATP (2.5 mM, 1 h), or the cells were treated with etoposide (Etopo, 200 μM, 16 h). The cells were stained with anti-CD45 antibody and PI, and analyzed by flow cytometry. (**b**) Representative immunofluorescence images of co-cultured *Nlrp3*$^{+/+}$ or *Nlrp3*$^{-/-}$ microglia and SH-SY5Y cells treated with MPTP (40 μM, 16 h), followed by treatment with ATP (2.5 mM, 15 min) after staining with anti-Iba1 (green) and anti-TH (red) antibodies. DAPI represents the nuclear signal (blue). Scale bars, 50 μm.

a

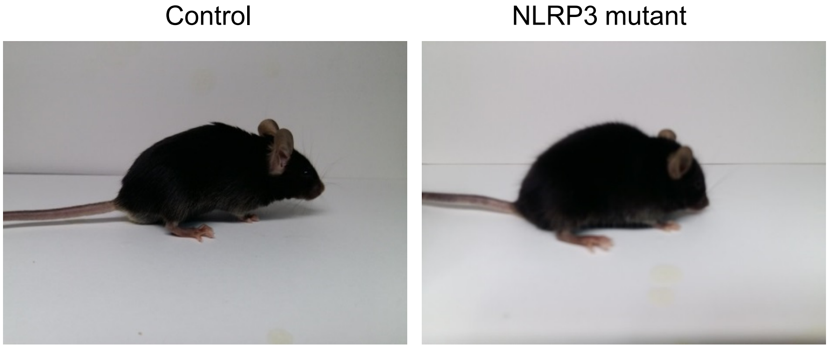

b

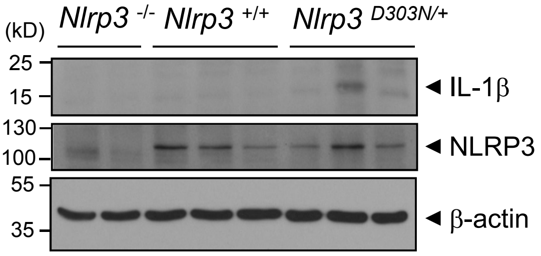

Supplementary Figure 8. (**a**) Control (*Cx3Cr1*$^{Cre\text{-}ER/Cre\text{-}ER}$*Nlrp3*$^{+/+}$) or NLRP3 mutant mice (*Cx3Cr1*$^{Cre\text{-}ER/+}$*Nlrp3*$^{D301NneoR/+}$) were treated with tamoxifen for 5 days, followed by the administration of MPTP for additional 3 days. Nlrp3 mutant mice showed more severe truncal dystonia. (**b**) Immunoblots from the substantia nigral extracts of MPTP-treated (5 days) *Nlrp3* $^{+/+}$ or *Nlrp3* $^{-/-}$ mice, or tamoxifen-treated *Nlrp3* $^{D303NneoR/+}$ mice upon MPTP administration (3 days) with the indicated antibodies.
