# Supplementary material for: MPTP-driven NLRP3 inflammasome activation in microglia plays a central role in dopaminergic neurodegeneration
Source: Cell Death Differ. 2018 May 21;26(2):213–28. doi: 10.1038/s41418-018-0124-5 (PMC6329843; doi:10.1038/s41418-018-0124-5)
Supplement: Supplementary file 2 — Supplementary discussion [file 41418_2018_124_MOESM2_ESM.doc]

**Supplementary Discussion**

It has been generally considered that NLRP3 inflammasome activation requires both signal 1 stimulus for the toll-like receptor mediated transcription and priming and signal 2 stimulus for the activation of NLRP3[^1^](#_ENREF_1). Our data clearly indicate that MPTP or MPP^+^ treatment could promote caspase-1 activation only in the presence of ATP or nigericin treatment. In this regard, MPTP or MPP^+^ might function as the signal 1 stimulus in our cell culture systems. Additionally, MPTP treatment clearly induced the robust expression of pro-IL-1β in mixed glial cell cultures. However, MPTP did not increase the expression of NLRP3 mRNA in mixed glial cells. Additionally, MPTP treatment failed to induce the phosphorylation of ERK and JNK, the possible priming events involved in the activation of NLRP3 inflammasome. On the other hand, MPTP or MPP^+^ stimulation did not induce K^+^ efflux, an essential signal 2-triggered intracellular phenomenon for NLRP3 activation. Of notice, although MPP^+^ impairs mitochondrial respiration leading to the mtROS production[^2^](#_ENREF_2), it did not directly cause mitochondrial spatial rearrangement into peri-nuclear regions, which is also driven by signal 2, in our study. These results suggest that MPTP or MPP^+^ is likely to provide a priming signal for NLRP3 activation, but the role of MPTP in the activation of NLRP3 requires further clarification at the molecular level.

**Supplementary References**

1. Haneklaus M, O'Neill LA, Coll RC. Modulatory mechanisms controlling the NLRP3 inflammasome in inflammation: recent developments. *Curr Opin Immunol* 2013; **25**: 40-45.

2. Cassarino DS, Parks JK, Parker WD, Jr., Bennett JP, Jr. The parkinsonian neurotoxin MPP+ opens the mitochondrial permeability transition pore and releases cytochrome c in isolated mitochondria via an oxidative mechanism. *Biochim Biophys Acta* 1999; **1453**: 49-62.
